# Rates and causes of 30-day readmission and emergency room utilization following head and neck surgery

**DOI:** 10.1186/s40463-018-0283-x

**Published:** 2018-05-18

**Authors:** Vincent Wu, Stephen F. Hall

**Affiliations:** 1 School of Medicine, Faculty of Health Sciences, Queen’s University, 80 Barrie St, Kingston, ON K7L 3J8 Canada; 2 Department of Otolaryngology – Head and Neck Surgery, Queen’s Cancer Research Institute, Queen’s University, 10 Stuart St, Level 2, Kingston, ON K7L 3N6 Canada

**Keywords:** Rates, Causes, 30-day, Readmission, Emergency department, Otolaryngology, Head and neck surgery

## Abstract

**Background:**

Unplanned returns to hospital are common, costly, and potentially avoidable. We aimed to investigate and characterize reasons for all-cause readmissions to hospital as in-patients (IPs) and visits to the Emergency Department (ED) within 30-days following patient discharge post head and neck surgery (HNS).

**Methods:**

Retrospective case series with chart review. All patients within the Department of Otolaryngology – Head and Neck Surgery who underwent HNS for benign and malignant disease from January 1, 2010 to May 31, 2015 were identified. The electronic medical records of readmitted patients were reviewed for reasons of readmission, demographic data, and comorbidities.

**Results:**

Following 1281 surgical cases, there were 41 (3.20%) IP readmissions and 109 (8.43%) ED visits within 30-days after discharge for HNS. For IP readmissions, most common causes included infection (26.8%), respiratory symptoms (17.1%), and pain (17.1%). Most common reasons for ED visits were for pain (31.5%), bleeding (17.6%), and infection (14.8%). Readmitted IPs had significantly higher health burden at pre-operative baseline as compared to patients who visited the ED when assessed with the American Society of Anesthesiology scores (*p* = 0.002) and the Cumulative Illness Rating Scale (*p* = 0.004).

**Conclusion:**

Rate of 30-day IP readmission and ED utilization was 3.20 and 8.43%, respectively. Pain and infection were common causes for returns to hospital. Discharge planning may be improved to target common causes for post-surgical hospital visits in order to decrease readmission rates.

## Background

Unplanned returns to hospital, including readmissions as in-patients (IPs) and visits to the Emergency Department (ED) are identified as costly, common, and potentially avoidable with proper planning and patient education [1–6]. The Canadian Institute for Health Information (CIHI) identified 30-day readmission rates to be a measure of a hospital’s quality and patient care [6]. From its 2012 report, surgical patients were identified as having the second highest overall rate of readmission as IP and return to the ED, contributing to the $1.8 billion dollar annual cost associated with 30-day readmissions in Canada [6]. While studies having indicated that between 9 to 59% of all unplanned readmissions are potentially avoidable, identifying these causes are necessary in order to reduce rates and the associated healthcare spending [5, 7–10].

In general, it is known that readmitted patients are older and have more medical comorbidities [11–13]. Being from a low socioeconomic class and having had previous unplanned visits to hospital also increase the risk for readmissions [11, 12, 14]. Moreover, the causes for readmission differ based on the specific patient population [15, 16]. Studies on surgery patients have identified risk factors for unplanned readmission that differ from medical patients [16]. Within the field of Otolaryngology – Head and Neck Surgery, the surgical procedures performed are diverse in nature. A single readmission rate is not representative of the specialty as a whole, and can range from 2 to 8% depending on the subspecialty focus [17, 18]. Patients who undergo head and neck surgery (HNS) have been shown to have higher rates of 30-day ED utilization and readmission as IPs [18–20].

Causes for unplanned readmission following HNS have not been thoroughly identified within a Canadian population. Our objective was to analyze and characterize the rates and reasons for all-cause readmission as IP and visits to the ED within 30-days following HNS. The results may serve to potentially reduce readmission rates by acting on preventable causes.

## Methods

### Study design and population

Retrospective chart review was performed of patients who were readmitted as IP or visited the ED within 30-days following discharge post-HNS from a tertiary academic center from January 1, 2010 to May 31, 2015. Patients were identified using CIHI procedural codes from hospital-based datasets. Patient cases were separated based on the procedure performed, which were separated into procedural categories. Examples of surgeries performed with procedural categories are shown in Table 1. Procedures pertaining to the nasal cavity, paranasal sinuses, skull base, ears, tonsils, adenoids or skin were not included.

**Table 1:** Included procedural categories

Procedure Categories	Example
Major Head and Neck with No Flap	Laryngectomy without flap reconstruction
Major Head and Neck with Pedicled Flap	Oropharyngeal resection with pectoralis myocutaneous rotation flap reconstruction
Major Head and Neck with Free Flap	Excision of oropharynx with radial forearm free flap reconstruction
Open Airway	Tracheostomy
Limited Oral Cavity	Marginal mandibulectomy
Limited Neck	Branchial cleft cyst resection
Neck Dissection Only	Cervical lymph node dissection
Salivary Gland	Parotidectomy
Thyroid/Parathyroid	Total thyroidectomy

### Definition and study variables

We defined 30-day readmission as an IP admission to hospital for any cause, regardless of assignment under the Department of Otolaryngology – Head and Neck Surgery or another clinical service, within 30-days following initial post-HNS discharge. ED utilization was defined as any visit to the ED within 30-days following post-HNS discharge.

The primary outcome measure was causes for 30-day IP readmission, extracted as the primary diagnosis from electronic hospital discharge summaries. Secondarily, we evaluated causes for ED utilization, extracted from ED charts as the final diagnosis. If more than one ED visit or IP admission was noted within the 30-day period following surgery, only the first readmission/visit was recorded.

Additionally, patient demographics including age and sex were captured. The American Society of Anesthesiologists (ASA) score, admission date, procedure date, initial discharge date, and return date were also included. Patient comorbidities were captured using the Cumulative Illness Rating Scale (CIRS), which is a validated summative index aimed at quantifying the overall physical impairment of the patient through 13 independent organ system domains, with higher scores indicating greater comorbid illness [21].

### Statistical analysis

All statistical analyses were performed using Prism (v7.0, GraphPad, La Jolla, CA, USA) and statistical significance was set to α = 0.05. Results are reported as mean ± standard deviation (SD). Standard descriptive statistics were used to characterize causes for readmission. Patient information including age, gender, sex, comorbidities, ASA score were also described using descriptive statistics. Student’s t-tests were performed to compare differences in ASA and CIRS among IPs and ED patients.

## Results

Of the 1281 patients who underwent HNS during the study period, 120 (9.37%) patients returned to hospital within 30-days of discharge. In total, there were 41 (3.20%) IP readmissions and 108 (8.43%) ED visits. There were 29 patients who were admitted as IPs through the ED. Patient demographics are summarized in Table 2.

**Table 2:** Baseline Patient Characteristics

Variable	Included (*n* = 120)
Age (mean ± SD years)	57.5 ± 20.7
Male (*n*, %)	78, 65%
Length of stay (mean ± SD days)	7.58 ± 17.1
CIRS (mean ± SD)	5.15 ± 3.44
ASA (mean ± SD)	2.82 ± 0.83
Previous radiotherapy (*n,* %)	9, 7.5%
Previous chemotherapy (*n,* %)	2, 1.7%
Previous chemoradiation therapy (*n,* %)	7, 5.8%

*SD – standard deviation

Causes for readmission to hospital as IPs are listed in Table 3. The single most common reason for IP readmission was for infection (26.8%), which included sepsis, surgical site infection, and urinary tract infection. Reasons for visiting the ED are listed in Table 4, with the most common cause being pain at the surgical site (31.5%).

**Table 3:** Rates and causes of in-patient admissions

Causes	Numbers	Rates
Infection: UTI, sepsis	11	26.83%
Respiratory: COPDE, dyspnea	7	17.07%
Pain: surgical/graft site	7	17.07%
Systemic: dehydration	7	17.07%
Neurologic: seizures	3	7.31%
Bleeding: surgical site	3	7.31%
Exacerbation of chronic condition	2	4.87%
Cardiac: chest pain	1	2.43%

*UTI – urinary tract infection; COPDE; chronic obstructive pulmonary disease exacerbation

**Table 4:** Rates and causes of Emergency Department visits

Causes	Numbers	Rates
Pain: surgical/graft site	34	31.48%
Bleeding: surgical/graft site	19	17.59%
Infection: surgical/graft site, UTI	16	14.81%
Equipment: nasogastric tube, tracheostomy tube, surgical drain	8	7.41%
Cardiac: chest pain, syncope	7	6.48%
Respiratory: dyspnea	7	6.48%
Gastrointestinal: nausea/vomit, constipation	6	5.56%
Neurologic: seizure, weakness	5	4.63%
Exacerbation of chronic condition	4	3.70%
Psychiatric: delirium	2	1.85%

*UTI – urinary tract infection

The CIRS comorbidity score in patients who were readmitted as IPs (6.29 ± 3.27) were significantly higher than patients who visited the ED (4.56 ± 3.39), *p* = 0.004. This was similarly seen with the ASA score, with readmitted IPs (3.12 ± 0.64) having significantly higher scores compared to patients visiting the ED (2.66 ± 0.88), *p* = 0.002. A simple linear regression of ASA and CIRS scores revealed a significant positive correlation between the measures, *F*(1,118) = 73.37, *p* < 0.001, with an *r*^2^ = 0.383.

## Discussion

This descriptive study highlighted the rates and causes of 30-day readmission following HNS for benign and malignant causes within a Canadian tertiary academic center. Although 30-day readmission rates have been used as a quality metric for hospital care, there are limitations to its use. While potentially preventable causes for readmission exist, there are factors which are non-modifiable that can contribute to patient readmission and hospital utilization. These factors include patient gender, race, socioeconomic status, and comorbidities [15]. Causes of readmission also differ based on the specific patient population. Therefore, by examining a subspecialty population such as patients who underwent HNS, the specific needs of that group may be identified. Although truly preventable causes of 30-day readmission are low given various non-modifiable factors, this study ultimately identified common reasons for hospital visits such as pain and infection, which we believe may be preventable with changes made to discharge planning and improved patient education.

The rate of IP readmission among post-HNS patient was found to be 3.20% within our cohort. This is below the 5.1–14.5% readmission rate currently reported for HNS patients in the United States [19, 20, 22]. Compared to the study by Bur et al. who examined risk factors and causes for readmissions within HNS for malignant causes, our IP readmission numbers were lower [19]. This may be due to the incorporation of patients undergoing HNS for benign causes within our study. Similar causes for readmissions were noted within our cohort, with infection being the most common, followed by respiratory causes and dehydration [19]. In terms of risk factors, Bur et al. noted the presence of medical comorbidities such as diabetes and dyspnea at baseline were associated with increased readmission [19]. Although we did not capture specific comorbidities, we noted that increased ASA and CIRS were higher for IP readmitted patient as compared with patients who visited the ED only. Together, our results reaffirm that infectious and respiratory symptoms are common causes for readmission, and that readmitted IPs patients have increased baseline disease burden and comorbidities. Furthermore, our results revealed potentially preventable causes for readmission such as dehydration, which may be linked to altered diets, inadequate pain control, and/or poor oral intake, all of which may be optimized prior to discharge.

Literature surrounding ED utilization have mainly focused on specific procedures, not HNS as a whole. One study reported ED utilization after thyroidectomy and parathyroidectomy to be 11.22%, with common causes being paresthesia and wound complications [23]. Within our cohort, we noted an ED utilization rate of 8.43%, with the most common cause due to pain. To us, this represented a preventable cause of hospital utilization that may be better improved with discharge planning and patient education. As discharge is a transition time from hospital to home, many patients may feel inadequately prepared based on the information received in hospital [24]. Specific interventions including early discharge planning and individualized education can potentially reduce readmissions and ED utilization by 75% [25–27]. Moreover, noting the pain trajectories and addressing those patients with high pain levels prior to discharge may help to further decrease ED utilization [28]. Ultimately, systems analysis of discharge planning can be used to identify various steps associated with patient discharge including the delivery of information, and the type and amount of information that is delivered to the patient, in order to optimize the process.

ASA status has been reported to have a significant positive associated with higher readmission rates, and was among the variables strongly associated with predicting readmissions [13]. CIRS has also been used among HNS patients, with higher scores reflective of worsening health burden for an individual [21, 29]. This suggested that IPs were sicker and had increased health burden at baseline, which may have predisposed them to more frequent and serious medical complications requiring in-hospital admission. Often, procedures within HNS involve the resection of head and neck cancers. It is known that head and neck cancer patients have more medical comorbidities, often resulting from chronic exposures to risk factors such as tobacco and alcohol [30–33]. Therefore, it is not surprising that with potential increases in risk factor exposure that we noted higher ASA and CIRS scores among patients who were readmitted as IPs.

Currently, the distinction between surgical and medical causes for post-operative complications is still unclear. Even for surgical complications, there is still no agreed upon definition [34]. Common methods for categorizing post-operative complications, including the Clavien-Dindo Classification, makes no distinction made between medical and surgical causes [35]. There have been attempts to separate readmission based on surgical complications (bleeding, wound dehiscence, and surgical site infection) from other medical complications [36]. However, the inherent limitation of this approach is that the exacerbation of medical comorbidities, or development of new medical conditions, may be a result of the surgical stress. The standardization of post-operative complications will be helpful in distinguishing between complications as a result of the surgical procedure versus an exacerbation of a pre-existing medical condition due to the general stresses of surgery. This will be important for eliciting preventable surgical causes aimed at decreasing the overall readmission rate.

This study has potential limitations. First, inherent to retrospective chart reviews, there exists the possibility of selection bias. A preliminary list of patients who returned to hospital within 30-days following head and neck surgery was generated automatically through the hospital’s centralized patient information database. To ensure validity of the extracted data, individual chart review was conducted by the study author (V.W.) only after extensive training on the electronic medical record. The senior author (S.F.H.) oversaw the data extraction with periodic reviews. Additionally, information was not extracted for patients who did not have a 30-day readmission or ED visit, thereby preventing risk assessment and comparisons being drawn between this group and patients who had a readmission. Moreover, data was not available for the rates of ED and IP readmission in regional hospitals for the same procedures, precluding the ability to compare readmissions rates outside of our academic center. Our sample size was limited by some of these factors, and as such, an increased study size can potentially address some of this study’s limitations. Future prospective studies can also aim to account for these potential limitations and utilize additional metrics for hospital and patient quality of care including patient-reported outcomes and length of stay.

## Conclusion

The 30-day IP readmission rate for post HNS patients was 3.20% and the ED utilization rate was 8.43%. Pain and infection represented common causes for returns to hospital. Discharge planning may be improved to target common causes for post-surgical readmission as potential steps in decrease hospital readmission and ED visit rates.

## Abbreviations

ASA: American Society of Anesthesiologists
CIHI: Canadian Institute for Health Information
CIRS: Cumulative Illness Rating Scale
COPD: Chronic obstructive pulmonary disease
ED: Emergency Department
HNS: Head and neck surgery
IP: In-patient
SD: Standard deviation
